# Factors associated with unsatisfactory ratings among medical courses in the 2025 Brazilian National Medical Education Assessment Exam: a cross-sectional study

**DOI:** 10.1590/1806-9282.20260604

**Published:** 2026-10-05

**Authors:** Paola Soledad Mosquera, Alicia Matijasevich, Ivan Wilson Hossni Dias, Cristiane de Jesus Almeida, Mário César Scheffer

**Affiliations:** 1 Universidade de São Paulo, Faculdade de Medicina, Departamento de Medicina Preventiva, Programa de Pós-Graduação em Saúde Coletiva – São Paulo (SP), Brazil.

**Keywords:** Medical schools, Medical education, Academic performance, Educational measurement, Brazil

## Abstract

**OBJECTIVE:**

This study considers institutional and contextual characteristics in an analysis of factors associated with unsatisfactory ratings among medical courses in the 2025 Brazilian National Medical Education Assessment Exam.

**METHODS:**

We analyzed secondary data from participating medical courses, with an interest in unsatisfactory ratings (1 or 2 out of 5). To measure associations of unsatisfactory ratings with course characteristics (i.e., institution type, location, existence duration, applicant-vacancy ratio, student-faculty ratio), logistic regression models estimated crude and adjusted odds ratios, with 95%CIs.

**RESULTS:**

Of 350 courses (119 public, 231 private), one third (n=107) underperformed. More public (95.8%) than private (55.8%) courses performed satisfactorily. In the multivariate model, courses in the 2nd and 3rd quartiles of the applicant-vacancy ratio had lower odds of receiving an unsatisfactory rating (aOR 0.47; 95%CI 0.23–0.93 and aOR 0.32; 95%CI 0.15–0.67). Courses with less than 10 years of existence had higher odds of an unsatisfactory rating (aOR 2.13; 95%CI 1.01–4.47). Higher odds were observed in the North (aOR 5.41; 95%CI 1.70–17.18), whereas lower odds were found in the South (aOR 0.39; 95%CI 0.15–0.98), compared with the Southeast. Private courses had higher odds of an unsatisfactory rating (aOR 10.93; 95%CI 2.68–44.53).

**CONCLUSION:**

Results showed inequalities in medical course ratings by institution type, existence duration, applicant-vacancy ratio, and geographical location, indicating the need to improve regulatory policies and processes for evaluating medical education in Brazil.

## INTRODUCTION

Over 4,000 medical schools worldwide offer medical education^1^, reflecting diverse educational systems and healthcare needs. In Brazil, the number of medical programs increased from 252 to 494 over the past decade^2^, consolidating the country’s position as having the second most medical schools behind India^1^. However, course heterogeneity regarding admission criteria, infrastructure, and training duration, influenced by regional, political, and economic factors, has sparked global discussions about the capacity and quality of medical education^3,4^. The discourse considers the potential adverse implications for health systems, patient safety, and responses to population health needs.

The evaluation of institutional quality in medical education includes accreditation processes, management-related aspects, pedagogical design, the institutional infrastructure required for medical education^5^, and performance measurement systems based on standardized exams applied to students^6,7^. Additionally, faculty evaluations^8^, governance structures, educational resources^9^, and clinical practice environments^10^ are indirect indicators of institutional quality. Unsatisfactory results tend to indicate persistent structural weaknesses in medical courses that may require regulatory actions and educational improvement.

In Brazil, in 2025, the Ministry of Education (MEC) unified the assessment instruments of the National Student Performance Exam (ENADE) and the National Residency Exam (ENARE), giving rise to the Brazilian National Medical Education Assessment Exam (ENAMED)^11^, which provides a standardized assessment of course-level performance and supports the admission process for medical residency. Although ENAMED does not encompass all dimensions involved in the quality of medical education, it offers a national indicator for evaluating educational outcomes. Graduands and graduates of medical course participants in ENARE are eligible. Among the 350 medical course participants in the inaugural ENAMED, 107 underperformed, prompting the MEC to supervisory actions^12^.

Multilevel analysis using data from ENADE and the Higher Education Census (2016–2018) indicated that although individual student characteristics explained some of the variation in performance, course-related attributes significantly influenced the results^13^. Several factors have been identified as potential determinants of the performance of medical courses under ENADE, including institutional characteristics, training capacity-related aspects, human resource availability, and courses’ territorial context^14,15^. However, these factors are underexplored in an integrated manner, particularly in analyses that adopt the course as the research unit.

Given the substantial expansion of course offerings, particularly in the private sector, this study recognizes that understanding the factors associated with obtaining unsatisfactory course ratings is relevant to improving regulatory and evaluation strategies. The research aimed to analyze factors associated with unsatisfactory ENAMED 2025 ratings among medical courses, considering their institutional and contextual characteristics.

## METHODS

### Data sources and study variables

This cross-sectional study used secondary data from medical courses participating in the first ENAMED, with the medical course as the unit of analysis. Course information was obtained from the ENAMED 2025 database^11^ and the Higher Education Census (“Courses” module)^16^, both provided by the National Institute for Educational Studies and Research Anísio Teixeira (INEP), and from the National Register of Higher Education Courses and Institutions (e-MEC)^17^. Population estimates of municipalities housing medical schools were based on data from the Brazilian Institute of Geography and Statistics^18^.

The following course characteristics were analyzed: institution type (public vs. private); location (north, northeast, southeast, south, or central-west) and municipal population (>300,000 or <300,000 inhabitants); existence duration (≥21, 11–20, or ≤10 years); applicant-vacancy ratio (i.e., admission competition), estimated as the total number of applicants divided by the total number of vacancies in 2020, considering that ENAMED 2025 participants were admitted to medical courses that year; and student-faculty ratio, estimated based on the total number of enrollees and faculty in 2023, subject to the availability of these data at the time of analysis. This study classified courses offered free of charge as public and those that charged monthly fees as private, regardless of whether the latter were subsidized. This operational classification was adopted because the primary interest was not the legal nature of the institution, but whether access to the course required tuition payment. Municipal population and existence duration were defined based on data or estimates corresponding to 2025, the ENAMED reference year.

The outcome of interest was whether a medical course received an unsatisfactory ENAMED 2025 rating. According to INEP methodology, course ratings are derived from the performance of graduating students with valid examination results, including the proportion achieving a proficient performance level. Courses with fewer than 10 valid participants do not receive a rating. The procedures used to estimate student proficiency and derive course ratings are described in official INEP technical documentation^19,20^. Ratings range from 1 to 5 and were grouped as unsatisfactory (1–2) and satisfactory (3–5)^19^.

### Statistical analysis

Course characteristics were described according to the ENAMED rating and type of the providing institution, considering absolute values (n) and relative frequencies (%). We compared course characteristics across ENAMED rating groups using Fisher’s exact and chi-square tests for categorical variables. The applicant-vacancy and the student-faculty ratios were asymmetrically distributed between courses and thus described by median and interquartile range (IQR: P25–P75) and compared using the Mann-Whitney test. To assess the association between course characteristics and unsatisfactory ENAMED ratings, logistic regression models estimated crude and adjusted odds ratios (aORs), with respective 95%CIs. In the adjusted model, all explanatory variables were included simultaneously based on their theoretical relevance and previous evidence indicating that institutional, contextual, and organizational characteristics may be associated with the performance of medical courses in educational assessments^14,15^. Given its asymmetric distribution and variability across courses, the applicant-vacancy ratio was categorized into quartiles for association analysis. After adjusting the models, we assessed the presence of multicollinearity among the explanatory variables using the Variance Inflation Factor (VIF). We also conducted a sensitivity analysis in which we estimated the model considering only courses rated 1 or 2 versus those rated 4 or 5 and excluding those rated 3. This verified whether inclusion of this category in the best performing group influenced the association estimates since a rating of 3 represents an intermediate performance.

For the analyses, we used Stata, version 16.0, and adopted a 5% significance level. This study was approved by the Research Ethics Committee of the Medical School of the University of São Paulo (CAAE No. 71626323.8.0000.0068). Artificial intelligence tools were used exclusively for linguistic revision and had no involvement in the generation of scientific content, data analysis, or the findings.

## RESULTS

Three hundred and fifty courses participated in ENAMED 2025, of which 119 and 231 were public and private, respectively (Table 1). Among them, 107 were rated as unsatisfactory. Most public courses (95.8%) achieved a satisfactory rating, whereas 55.8% of private courses did. Indeed, 102 private courses were rated as unsatisfactory (1 or 2), whereas only five public courses were rated as underperforming.

**Table 1:** Characteristics of medical courses by National Medical Education Assessment Exam 2025 rating and institution type in Brazil (n=350 courses).

Medical courses	ENAMED 2025 rating								
	Total			Public courses			Private courses		
	1 and 2	3, 4, and 5	p-value^a^	1 and 2	3, 4, and 5	p-value^a^	1 and 2	3, 4, and 5	p-value^a^
Courses, n (%)	107 (30.6)	243 (69.4)	<0.001	5 (4.2)	114 (95.8)	0.103	102 (44.2)	129 (55.8)	<0.001
Applicant-vacancy ratio, median (IQR)	3.6 (1.9–6.8)	17.0 (5.8–65.9)		46.8 (40.5–53.2)	66.9 (44.7–116.0)		3.4 (1.9–6.2)	7.3 (3.8–13.0)	
Applicant-vacancy ratio, n (%)									
1st quartile (lowest admission competition)	52 (59.1)	36 (40.9)	<0.001	1 (11.1)	8 (88.9)	0.741	51 (64.6)	28 (35.4)	<0.001
2nd	33 (37.9)	54 (62.1)		0 (0.0)	2 (100.0)		33 (38.8)	52 (61.2)	
3rd	19 (21.6)	69 (78.4)		1 (4.4)	22 (95.6)		18 (27.7)	47 (72.3)	
4th quartile (highest admission competition)	3 (3.5)	84 (96.5)		3 (3.5)	82 (96.5)		0 (0.0)	2 (100.0)	
Student-faculty ratio^b^, median (IQR)	10.0 (7.1–14.6)	5.6 (4.0–7.9)	<0.001	8.7 (7.7–8.9)	4.5 (3.4–5.8)	<0.001	10.3 (6.9–14.7)	7.1 (5.1–10.8)	<0.001
Existence duration^c^, n (%)									
≥21 years	27 (19.2)	114 (80.8)	<0.001	0 (0.0)	63 (100.0)	0.014	27 (34.6)	51 (65.4)	0.001
11–20 years	27 (25.2)	80 (74.8)		3 (7.7)	36 (92.3)		24 (35.3)	44 (64.7)	
≤10 years	53 (52.0)	49 (48.0)		2 (11.8)	15 (88.2)		51 (60.0)	34 (40.0)	
Region, n (%)									
North	14 (46.7)	16 (53.3)	0.012	2 (14.3)	12 (85.7)	0.211	12 (75.0)	4 (25.0)	<0.001
Northeast	26 (30.2)	60 (69.8)		2 (4.8)	40 (95.2)		24 (54.6)	20 (45.4)	
Southeast	45 (31.9)	96 (68.1)		0 (0.0)	30 (100.0)		45 (40.5)	66 (59.5)	
South	8 (13.8)	50 (86.2)		1 (5.3)	18 (94.7)		7 (17.9)	32 (82.1)	
Central-West	14 (40.0)	21 (60.0)		14 (100.0)	14 (100.0)		14 (66.7)	7 (33.3)	
Location by municipal population size^d^, n (%)									
>300,000 inhabitants	44 (25.3)	130 (74.7)	0.033	1 (1.6)	61 (98.4)	0.192	43 (38.4)	69 (61.6)	0.087
<300,000 inhabitants	63 (35.8)	113 (64.2)		4 (7.0)	53 (93.0)		59 (49.6)	60 (50.4)	
Institution type, n (%)									
Public	5 (4.2)	114 (95.8)	<0.001						
Private	102 (44.2)	129 (55.8)							

Source: Authors; National Institute for Educational Studies and Research Anísio Teixeira/Ministry of Education. ENAMED: National Medical Education Assessment Exam; IQR: interquartile range. Note: In 2020, a medical course failed to respond to the Higher Education Census; we obtained its data from the 2021 census.

^a^p-values estimated using Mann-Whitney, Fisher’s exact, and chi-squared tests, as appropriate.

^b^Student-faculty ratio was based on information from the 2023 Higher Education Census.

^c^Course existence duration is the period between its inaugural year and 2025.

^d^Population estimates for 2025 were obtained from the Brazilian Institute of Geography and Statistics.

The applicant-vacancy ratio was significantly lower among underperforming courses (median: 3.6; IQR: 1.9–6.8) than among those with a satisfactory rating (median: 17.0; IQR: 5.8–65.9). Public courses had a higher applicant-vacancy ratio than private courses. Competition for admission to private courses was significantly lower among those with an unsatisfactory rating (median: 3.4 vs. 7.3). The student-faculty ratio was significantly higher among courses with an unsatisfactory rating (median: 10.0; IQR: 7.1–14.6) than among those with a satisfactory rating (median: 5.6; IQR: 4.0–7.9). Public courses had a lower student-faculty ratio than private courses. In both types of institution, courses with an unsatisfactory rating had more students per faculty member than courses with a satisfactory rating.

The proportion of courses with an unsatisfactory rating increased as existence duration decreased, with 19.2% of courses operating for 21 years or more, 25.2% of those operating for 11–20 years, and 52.0% of those operating for up to 10 years receiving unsatisfactory ratings. Conversely, the proportion of courses with a satisfactory rating was higher among older programs. No public course in existence for 21 years or more (n=63) received an unsatisfactory rating, but 11.8% (n=2) of those less than 10 years old (n=17) did. Among the oldest private courses, 34.6% were rated 1 or 2, and 65.4% were rated 3–5.

The proportion of underperforming courses was 25.3% in schools located in populous municipalities and 35.8% in those in smaller municipalities (p=0.033). No significant differences were observed by institution type after stratification. The south had the lowest proportion of underperforming courses (13.8%), followed by the northeast (30.2%), southeast (31.9%), central-west (40%), and north (46.7%; Table 1).

The multivariate model revealed an association of applicant-vacancy ratio, existence duration, location, and institution type with courses’ ENAMED ratings (Table 2). As the applicant-vacancy ratio increased, the odds of receiving an unsatisfactory rating decreased. Compared to the first quartile comprising institutions with the lowest applicant-vacancy ratio, the second and third quartiles showed 53% (aOR 0.47; 95%CI 0.23–0.93) and 68% (aOR 0.32; 95%CI 0.15–0.67) reductions in the odds of an unsatisfactory rating, respectively. Courses operating for less than 10 years had higher odds of receiving an unsatisfactory rating than those operating for 21 years or more (aOR 2.13; 95%CI 1.01–4.47). Courses offered in the North had higher odds of receiving an unsatisfactory rating (aOR 5.41; 95%CI 1.70–17.18), whereas those in the South had lower odds (aOR 0.39; 95%CI 0.15–0.98) than those in the Southeast. Private courses had higher odds of being rated as unsatisfactory than public courses (aOR 10.93; 95%CI 2.68–44.53). The 95%CI was wide, possibly due to the small number of events among public courses. No evidence of problematic multicollinearity (mean VIF=1.69) was observed among the independent variables included in the adjusted model.

**Table 2:** Adjusted logistic regression analysis of factors associated with an unsatisfactory rating in National Medical Education Assessment Exam in Brazil (n=350 courses).

Course characteristics	ENAMED rating of 1 or 2			
	OR (95%CI)	p-value	aOR (95%CI)	p-value
Applicant-vacancy ratio				
1st quartile (lowest admission competition)	1	<0.001	1	0.011
2nd	0.42 (0.23–0.77)		0.47 (0.23–0.93)	
3rd	0.19 (0.10–0.36)		0.32 (0.15–0.67)	
4th quartile (highest admission competition)	0.02 (0.01–0.08)		0.21 (0.03–1.18)	
Student-faculty ratio	1.09 (1.05–1.13)	<0.001	1.02 (0.99–1.06)	0.138
Existence duration				
≥21 years	1	<0.001	1	0.050
11–20 years	1.42 (0.77–2.61)		0.90 (0.43–1.86)	
≤10 years	4.5 (2.57–8.08)		2.13 (1.01–4.47)	
Region				
North	1.86 (0.83–4.15)	0.017	5.41 (1.70–17.18)	0.002
Northeast	0.92 (0.51–1.65)		1.64 (0.78–3.42)	
Southeast	1		1	
South	0.34 (0.14–0.77)		0.39 (0.15–0.98)	
Central-West	1.42 (0.66–3.05)		1.97 (0.72–5.38)	
Location by municipal population size				
>300,000 inhabitants	1	0.034	1	0.305
<300,000 inhabitants	1.64 (1.03–2.60)		1.39 (0.73–2.63)	
Institution type				
Public	1	<0.001	1	0.001
Private	18.02 (7.09–45.80)		10.93 (2.68–44.53)	

ENAMED: National Medical Education Assessment Exam; aOR: adjusted odds ratio; CI: confidence interval; OR: odds ratio. Note: Variance inflation factors (VIFs) for the adjusted model indicated no evidence of problematic multicollinearity (mean VIF=1.69; range: 1.16–3.39).

The sensitivity analysis restricted to comparing courses rated 1 or 2 with those rated 4 or 5 yielded results consistent with the main model, with no change in the direction of the associations. Although some 95%CIs were wider because of the reduced sample size, the overall pattern of findings remained unchanged (Table 3).

**Table 3:** Adjusted logistic regression of factors associated with an unsatisfactory rating in National Medical Education Assessment Exam 2025, comparing scores 1–2 versus 4–5, Brazil (n=270).

Course characteristics	ENAMED rating of 1 or 2			
	OR (95%CI)	p-value	aOR (95%CI)	p-value
Applicant-vacancy ratio				
1st quartile (lowest admission competition)	1	<0.001	1	0.001
2nd	0.42 (0.23–0.77)		0.38 (0.14–1.02)	
3rd	0.19 (0.09–0.36)		0.15 (0.05–0.41)	
4th quartile (highest admission competition)	0.02 (0.01–0.08)		0.11 (0.02–0.69)	
Student-faculty ratio	1.09 (1.05–1.13)	<0.001	1.03 (0.98–1.09)	0.181
Existence duration				
≥21 years	1	<0.001	1	0.028
11–20 years	1.42 (0.77–2.61)		1.13 (0.47–2.72)	
≤10 years	4.56 (2.57–8.09)		3.56 (1.33–9.52)	
Region				
North	1.86 (0.83–4.15)	0.017	11.89 (2.11–67.05)	0.007
Northeast	0.92 (0.51–1.65)		1.75 (0.63–4.86)	
Southeast	1		1	
South	0.34 (0.14–0.77)		0.33 (0.10–1.01)	
Central-West	1.42 (0.66–3.05)		1.45 (0.38–5.56)	
Location by municipal population size				
>300,000 inhabitants	1	0.034	1	0.288
<300,000 inhabitants	1.64 (1.03–2.60)		1.55 (0.69–3.48)	
Institution type				
Public	1	<0.001	1	<0.001
Private	18.02 (7.09–45.80)		25.27 (5.33–119.67)	

ENAMED: National Medical Education Assessment Exam; aOR: adjusted odds ratio; CI: confidence interval; OR: odds ratio.

The results of ENAMED 2025 revealed that 30% of participating courses underperformed. Private courses, those operating for less than 10 years, those with a lower applicant-vacancy ratio, and those offered in the North had higher odds of receiving an unsatisfactory rating.

Prior studies conducted based on ENADE anticipated worse results, on average, for private (vs. public) medical schools^21,22^. The hegemony of private medical schools in Brazil (where 72% of all medical courses in 2024 were private)^2^ was accompanied by a significant increase in the offering of physician services and the relative geographical deconcentration of professionals^23^. However, the present findings indicate substantial heterogeneity within the private sector, with a considerable proportion of private courses receiving unsatisfactory ENAMED ratings (44.2%). These results suggest that some private medical courses may require closer regulatory monitoring and targeted quality-improvement strategies.

In line with our results, previous studies have demonstrated better performance of courses in capital cities^13^, as well as those in the south, and worse performance of courses in the north^14^. International evidence has shown that medical schools and other factors may contribute to the establishment of physicians in underserved regions and/or those that are remote from big cities^23^. However, regional differences may reflect broader inequalities in educational and healthcare infrastructure, availability of clinical training environments, and faculty recruitment and retention. The regionalization of medical education remains an important strategy for expanding access to training and addressing workforce shortages, but it should be accompanied by adequate investments and quality-monitoring mechanisms. Further studies are needed to assess whether limitations in infrastructure, clinical training environments, and faculty availability are related to poor ENAMED performance.

This study found an association of greater admission competition (applicant-vacancy ratio) with a lower chance of an unsatisfactory rating in ENAMED. Medicine is among the undergraduate courses with the highest admission competition in Brazil, but the applicant-vacancy ratio varies widely among institutions. The literature identifies the diversity of admission processes and recruitment formats based on standardized tests, previous school performance, affirmative policies, and other factors as a methodological challenge to analyzing the relationship between admission and academic performance^24^. In this context, discussing the improvement of admission mechanisms in medical schools is necessary, with consideration to both performance in evaluations and the training of professionals to foster their development of capabilities appropriate to medical practice.

Relatively new medical courses performed poorly in ENAMED. Many were authorized in the last decade through expansion policies such as the Lei Mais Médicos (More Physicians Act 2013)^25^ and, in some cases, by court decisions that required the MEC to process opening requests. Shorter existence duration may reflect lower institutional maturity, including less consolidated educational projects, faculty structures, clinical training environments, and integration with health services. These findings reinforce the importance of not only meeting initial requirements for course authorization but also ensuring adequate conditions for sustaining educational quality over time.

Overall, the findings were robust across sensitivity analyses. The exclusion of courses with concept 3 yielded consistent estimates, with no changes in the direction of associations, although with wider confidence intervals.

This study has limitations. Its cross-sectional design does not allow causal inferences. The analysis relied on secondary databases and used the medical course as the unit of analysis, precluding individual-level interpretations. In addition, relevant institutional characteristics, such as clinical training environments, preceptorship, and teaching-service integration, were unavailable. Furthermore, the classification of institutions as public or private may not fully capture the heterogeneity of the private sector, potentially introducing residual confounding. Finally, estimates related to institution type should be interpreted cautiously due to the small number of unsatisfactory ratings among public courses, which may have reduced the precision and stability of the corresponding estimates.

## CONCLUSION

Approximately one third of ENAMED 2025 courses received an unsatisfactory rating. Private institutions, shorter course existence duration, less competitive admission processes (lower applicant-vacancy ratios), and location in the North region were associated with this outcome. These findings may help strengthen regulatory policies in Brazil and identify courses that warrant closer monitoring and targeted quality-improvement strategies, although ENAMED results should be interpreted alongside other indicators of medical education quality.

## Data Availability

The original datasets used in this study are publicly available from their respective sources. The merged analytical dataset, including derived variables created for the analyses, is available from the corresponding author upon reasonable request.
